# The Eyes

**Published:** 1877-07

**Authors:** 


We urge upon all parents, in view of the many incurable eye diseases, to caution their children against reading by twilight, that is, not before sunrise nor after sunset. It would be greatly better not to allow them to read or sew by any artificial light; but if that is unavoidable, let it be imperative that they cease by nine o'clock at night in summer, and by ten at farthest, in the winter. It is a most inexcusable folly, and will, sooner or later, bring its punishment, to read or sew by gas, or lamp, or candle light, and then sleep after daylight next morning, as a habit. To persons of all ages it is a most injurious practice.

